# Design Principles From Natural Olfaction for Electronic Noses

**DOI:** 10.1002/advs.202412669

**Published:** 2025-01-21

**Authors:** Haritosh Patel, Vicente Garrido Portilla, Anna V. Shneidman, Jacopo Movilli, Jack Alvarenga, Christophe Dupré, Michael Aizenberg, Venkatesh N. Murthy, Alexander Tropsha, Joanna Aizenberg

**Affiliations:** ^1^ Harvard John A. Paulson School of Engineering and Applied Sciences Harvard University Boston MA 02134 USA; ^2^ Department of Chemical Sciences University of Padova Padova 35131 Italy; ^3^ Department of Molecular & Cellular Biology Harvard University Cambridge MA 02138 USA; ^4^ Center for Brain Science Harvard University Cambridge MA 02138 USA; ^5^ Kempner Institute Harvard University Boston MA 02134 USA; ^6^ Department of Chemistry The University of North Carolina at Chapel Hill Chapel Hill NC 27516 USA; ^7^ Department of Chemistry and Chemical Biology Harvard University Cambridge MA 02138 USA

**Keywords:** adaptive mechanisms, bioinspiration, gas sensors, machine learning, materials science

## Abstract

Natural olfactory systems possess remarkable sensitivity and precision beyond what is currently achievable by engineered gas sensors. Unlike their artificial counterparts, noses are capable of distinguishing scents associated with mixtures of volatile molecules in complex, typically fluctuating environments and can adapt to changes. This perspective examines the multifaceted biological principles that provide olfactory systems their discriminatory prowess, and how these ideas can be ported to the design of electronic noses for substantial improvements in performance across metrics such as sensitivity and ability to speciate chemical mixtures. The topics examined herein include the fluid dynamics of odorants in natural channels; specificity and kinetics of odorant interactions with olfactory receptors and mucus linings; complex signal processing that spatiotemporally encodes physicochemical properties of odorants; active sampling techniques, like biological sniffing and nose repositioning; biological priming; and molecular chaperoning. Each of these components of natural olfactory systems are systmatically investigated, as to how they have been or can be applied to electronic noses. While not all artificial sensors can employ these strategies simultaneously, integrating a subset of bioinspired principles can address issues like sensitivity, drift, and poor selectivity, offering advancements in many sectors such as environmental monitoring, industrial safety, and disease diagnostics.

## Graphical abstract

Unlocking “universal smell” requires integrating key principles of natural olfactory systems: modulation of gas transport, utilization of non‐selective sensing elements, chemical filters akin to the biological mucus layer to separate compounds, refined context‐specific signal integration, and more. These components work in harmony to improve the accuracy, sensitivity, and adaptability of electronic noses whilst mitigating common failure modes.

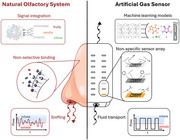

## 1 Introduction

The need to detect specific gases is critical across a wide range of industries, from breath analysis for disease diagnosis to hazardous waste identification and classification.^[^ ^1^, ^2^ ^]^ The standard approach has been to use bulky, expensive equipment (e.g., infrared spectroscopy, gas chromatography, and mass spectroscopy), typically requiring trained personnel and, for many applications, necessitating off‐site analysis.^[^ ^3^ ^]^ Over the last century, this has motivated the development of alternative technologies to detect volatile molecules in the form of small easy‐to‐use devices, allowing for real‐time measurements and immediate responses. Toward this goal, researchers have sought reliable methods of transducing the presence of a volatile chemical into an interpretable signal, akin to how biological sensors, noses (or antennae in insects, antennules in crustaceans, etc.), convert binding events between odorants and odorant receptors (ORs) into action potentials, the neuronal impulses that ultimately reach the brain for processing and output. Beginning in the 1950s, much of the work centered on developing materials whose interaction with volatiles could produce measurable signals, such as changes in electrical or optical readouts, mechanical resonance shifts, or other mechanisms.^[^ ^4^ ^]^ The pioneering 1982 paper by Persaud and Dodd introduced the “electronic nose” (e‐nose), the concept of using an array of sensors, each of which can interact with multiple analytes with different strengths.^[^ ^5^ ^]^ This directly mimics the array concept implemented across biological olfactory systems, where odorants loosely bind multiple ORs and each OR is sensitive to many odorants. Advancements in materials fabrication, electronics, signal processing, machine learning (ML), and computer hardware for handling large datasets have significantly enhanced e‐nose performance^[^ ^6^, ^7^ ^]^, leading to their commercialization beginning in the 1990s.^[^ ^8^, ^9^ ^]^ The persistent goal motivating these developments has been to achieve biology's remarkable ability to identify odorant mixtures or to pick out specific compounds with exceptional sensitivity, even in complicated and fluctuating environments. In parallel, understanding of the biological olfactory system has evolved over this period of time and continues to be an area of active study^[^ ^10^, ^11^ ^]^, largely aided by developments in imaging techniques, neural activity monitoring and control, behavioral studies, and computational modeling methods. These innovations provide high‐resolution views of the anatomy and physiology of the olfactory system and help identify and unravel the complexities of the neural circuitry governing signal transmission, modulation, and processing.

Despite some successes, e‐noses have yet to revolutionize gas sensing. They continue to suffer from limitations outlined in a 1994 review by Gardner and Bartlett^[^ ^12^ ^]^, including low sensitivity and selectivity, and high sensor drift; additional challenges include the scale‐up of emerging materials, the collection of sufficiently large datasets to train new algorithms, device miniaturization, portability, and reduction of power consumption.^[^ ^13^ ^]^ Current users of commercially available e‐noses across industries report unreliable measurements, the need for frequent, laborious, and time‐consuming calibrations, and limited useful information, for example, providing only an aggregate measurement of total volatile organic compounds (TVOCs) rather than identifying the chemical species of interest. These limitations are primarily driven by the complex binding kinetics of volatiles on the sensor surface, where factors like concentration‐dependent competitive binding and environmental interferences (e.g., temperature and humidity fluctuations) complicate accurate detection.^[^ ^14^, ^15^ ^]^

**Figure 1:**
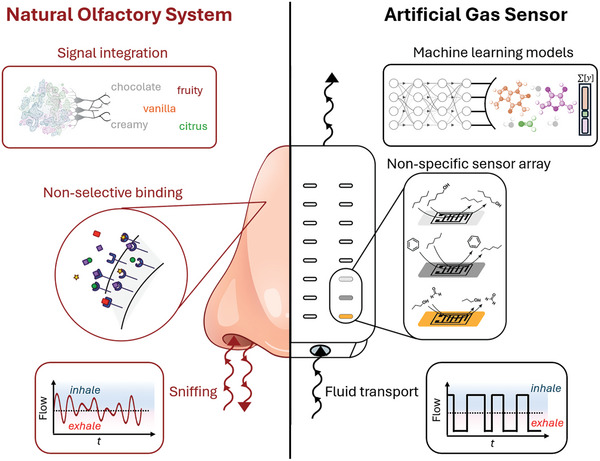
Principles of biological olfaction inform next‐generation gas sensor development. Unlocking “universal smell” requires integrating key principles of natural olfactory systems: modulation of gas transport, utilization of non‐selective sensing elements, and refined context‐specific signal integration. These components work in harmony to improve the accuracy, sensitivity, and adaptability of artificial sensors whilst mitigating common failure modes.

The history of e‐noses to date in both research and commercial spaces has been presented in recent comprehensive reviews.^[^ ^8^, ^9^, ^13^, ^16^, ^17^, ^18^, ^19^ ^]^ The purpose of this perspective is to examine the multitude of structures and phenomena that occur in the biological nose and to propose a holistic approach that fully embraces the principles of the natural olfactory system both in hardware and software, going beyond large sensor arrays (**Figure** **1**). While most of the examples are directly based on mammals or insects, many of the phenomena have close analogs in other animals. The presented principles can be applied across a wide range of existing and emerging e‐nose platforms, including those based on chemiresistive, nanomechanical, optical, catalytic mechanisms, and beyond. For example, chamber designs surrounding sensor elements have been inspired by the nasal anatomy to direct gas transport, potentially increasing the e‐nose sensitivity and discriminatory capability, while reducing the overall device size.^[^ ^20^, ^21^ ^]^ Likewise, the modulation of gas delivery to the sensing element—akin to sniffing—has been applied to enhance the performance of ML models for the detection and concentration quantification of a variety of vapor sources, such as solvents, beers, and household cleaning products.^[^ ^22^, ^23^, ^24^ ^]^ The ML models themselves have been designed to mimic the evermore‐sophisticated neuronal circuits being discovered in the biological olfactory system, including enhancing and suppressing signals based on gas mixture composition, and adapting to both fluctuating environments and sensor drift or even failure.^[^ ^12^, ^25^ ^]^

Each of the following sections examines a number of related biological principles, includes examples of where they have already been implemented, and provides a vision of how they can be implemented further individually or in concert to help overcome remaining challenges. Understanding the fluid mechanics of odorants as they move through natural channel geometries (Section 2), along with their intermolecular interactions with various parts of the olfactory system (Section 3), offers insights for designing hardware across different length and time scales, as well as for the chemical functionalization of surfaces in next‐generation gas sensing devices. Signal processing and analysis can be informed by the olfactory system's sophisticated spatiotemporal signal mapping, enabling the accurate identification and concentration measurement of volatiles (Section 4). The brain—and by extension, neural networks—also benefit from active modulation techniques, such as sniffing, which effectively enriches the information encoded in the signal (Section 5), and the translocation of the sensing system, involving changes in directionality and relative positioning of sensors (Section 6). Additionally, effects like biological priming and chaperoning offer intriguing opportunities for enhancing artificial olfaction, akin to the color complementarity observed in vision (Section 7). Importantly, olfactory systems offer remarkable adaptability, self‐optimization, and plasticity, which can be encoded in software or even in hardware by using environment‐responsive or self‐healing materials. Not all of these strategies need to be implemented simultaneously in all gas sensors, and subsets or combinations of these bioinspired principles may be particularly useful for specific real‐world tasks. As such, they provide a foundation for the development of new, more effective chemical gas sensors.

## 2 Nasal Anatomy Determines the Fluid Dynamics of Volatiles

For a volatile stimulus to be transduced into a signal interpretable by the brain, it must first be transported to the ORs, which are distributed in the olfactory epithelium located at the back of the nasal cavity. The shape of the nasal cavity and its internal structures can greatly influence the residence time of air near the olfactory epithelium, as demonstrated through experimental measurements and computational fluid dynamics (CFD) simulations.^[^ ^26^ ^]^

**Figure 2:**
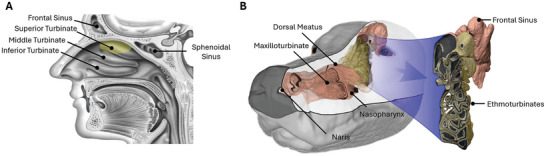
Nasal passageways of A) humans and B) dogs. In humans, the olfactory epithelium covers only the superior turbinate, while in dogs, it extends over both the superior turbinate and ethmoturbinates, enhancing their sense of smell. Images adapted with permission from ref. [26] Copyright 2009, The Royal Society.

In mammals, the nasal cavity consists of several distinct regions. Inhaled air first enters the nasal vestibule, where it encounters nasal hairs covered by mucus that acts as a filter, trapping pathogens and particulate matter.^[^ ^27^ ^]^ Further, highly vascularized, mucus‐covered curved bony structures, known as turbinates or conchae, partition the nasal cavity into distinct spaces. Humans have three turbinates, with the olfactory epithelium covering only the top space, including the upper surface of the superior turbinate (**Figure** **2**A).^[^ ^26^ ^]^ In contrast, dogs have a more complex turbinate structure, with additional ethmoturbinates located deeper within the nasal cavity. The olfactory epithelium in dogs extends over a larger area, covering both the superior and middle turbinate, parts of the ethmoturbinates (Figure 2B), contributing to their enhanced sense of smell.^[^ ^26^ ^]^ Turbinates are thought to serve three main purposes in odor sensing: i) directing the airflow, ii) increasing the surface area of interaction between odorants and ORs, such as the neuroepithelium located in the middle turbinates^[^ ^28^ ^]^, and iii) providing humidity and thermal conditioning of the incoming air to protect the delicate natural olfactory apparatus^[^ ^29^ ^]^; the latter is especially evident in animals endemic to Arctic regions, such as the bearded seal and reindeer.^[^ ^30^, ^31^, ^32^, ^33^, ^34^ ^]^

Dogs, rats, and other macroosmats (“super smellers”) have separate airflows for inhaled and exhaled air.^[^ ^26^, ^35^, ^36^ ^]^ Craven et al.’s CFD simulations of airflow in Labrador retrievers (*Canis familiaris*) revealed two types of laminar flow patterns during inhalation: i) flow directed toward the nasopharynx for respiration and ii) flow moving past the passage that houses the olfactory epithelium.^[^ ^26^ ^]^ As exhaled air in dogs does not pass through the olfactory recess, it does not significantly promote the desorption of odorants from the ORs. Odorants eventually desorb through diffusion or are actively removed by enzymatic metabolism or via mucociliary action; the latter is also responsible for clearing particulates.^[^ ^37^, ^38^ ^]^ These processes operate on time scales that allow for long enough odorant–OR residence times for sampling reliability and signal integration (i.e., in milliseconds to seconds range) without severely impeding the turnover required for detecting new odors. Prolonged interactions can also be achieved without the separation of the breathing and olfaction airways as well, such as in the bat species *Carollia perspicillata*, which exhibits a diminished outward airflow during exhalation due to the reduced size of their olfactory recess.^[^ ^39^, ^40^ ^]^ Although decoupling respiratory and olfactory airflow is not necessary for artificial sensor systems, more accurate, timely assessments of gas composition and concentration can be achieved through the design of sensor form factors that optimize the balance between the residence time (increasing signal) and the desorption rates (increasing turnover frequency).

Researchers are beginning to incorporate bioinspired geometric design principles into artificial sensors.^[^ ^20^, ^21^, ^41^, ^42^, ^43^, ^44^, ^45^, ^46^ ^]^ Lopez et al., for instance, report on the influence of chamber design (straight‐path vs U‐path) on the gas sensing performance of a graphene field‐effect transistor.^[^ ^21^ ^]^ By solving Navier–Stokes and convection‐diffusion equations using COMSOL, they confirmed that the gas flow direction through the chamber was a significant contributor to the sensor performance. Specifically, swirling vapor flow in the chamber brought about by the U‐shaped flow streamlines causes increased interaction of analyte molecules with the transistor surface, resulting in higher computed sensitivity. Chang et al. take further inspiration from the intricate nasal turbinates, demonstrating reduced airflow rates and increased turbulence, allowing them to scale down the size of their e‐nose while increasing the residence time of the analyte gases at the sensor array.^[^ ^20^ ^]^ Other implementations show promising increases in the limit of detection and selectivity, collectively highlighting the importance of fluid dynamics and chamber design in optimizing the performance of any e‐nose. Future developments in this space may include better separation of inlet and outlet feeds, turbulent mesoporous paths, or passive one‐way valves while maintaining a balance between benefits to sensor readout and manufacturability.

Further, for applications requiring real‐time monitoring, achieving both a high signal‐to‐noise ratio (SNR) and rapid sensor reset is essential, especially in high‐risk situations. Several approaches in this vein use effects largely beyond those found in biology, for example as demonstrated by Schüler et al., where temperature cycling of the integrated platinum heater, i.e., temperature cycle optimization (TCO), in a tin oxide gas sensor led to faster turnover between saturated signals, reduction of sensor poisoning, and improved overall performance.^[^ ^47^, ^48^, ^49^ ^]^ Such approaches can be coupled with ML algorithms incorporating feedback loops based on the sensor readouts to enable self‐monitoring and self‐adjusting in response to the detection of saturation events or prolonged adsorption. Recording the readouts at different temperatures can also enhance the information obtained from the sensors, improving classification, similar to the effects of gas modulation discussed in Section 5. Additional bioinspired designs include adjustable flow paths and dynamic filtering mechanisms that adapt to real‐time conditions that could further optimize the balance between analyte‐sensor interaction and turnover frequency. Moreover, tunable residence times can be achieved by incorporating other biological principles (rather than the non‐bioinspired temperature changes), including analogs to xenobiotic metabolizing enzymes and tunable odorant‐OR binding affinities, described in Sections 3 and 7, respectively. Such strategies allow sensors to maintain high sensitivity while providing the rapid response required for critical applications, thereby enhancing their performance and versatility across diverse environments.

## 3 Physicochemical Properties of Odorants Affect their Transport and Binding

The transport of an odorant to ORs is not only shaped by the physical structure of the nasal passageways but also by the odorant's physicochemical properties. An odorant's chemical structure determines its vapor pressure, diffusion coefficient, and air–water partition coefficient, ultimately affecting its distribution across media, including the air between the source and the nasal vestibule and the mucus covering the olfactory epithelium.^[^ ^50^, ^51^ ^]^ This influence is crucial during two stages: the initial movement of the odorant from its source to the nostrils and its subsequent diffusion within the mucus covering the olfactory epithelium.^[^ ^52^ ^]^ Odorants with higher volatility typically appear at higher concentrations at the entrance of the nose, though these levels can be modified by active inhalation, where other properties such as fluid viscosity, density, and vapor pressure become important. These physicochemical properties together with airflow patterns, humidity levels, and the unique anatomy of the nasal cavity ultimately determine the concentration of odorants at the ORs.^[^ ^53^, ^54^, ^55^ ^]^

**Figure 3:**
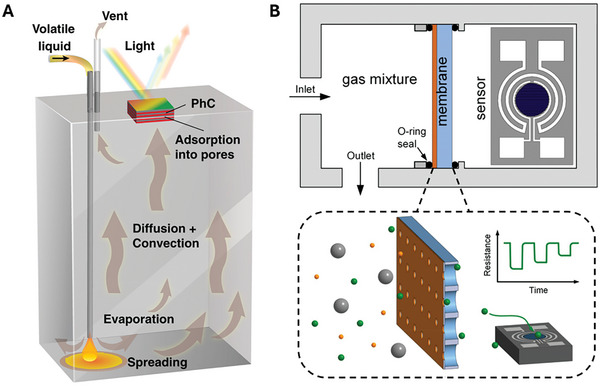
Strategies for odorant separation based on physicochemical properties. A) Spatial separation between the volatile source and the sensing element enables the differentiation of chemical compounds based on their dynamic transport behavior. PhC: Photonic Crystal. B) Microporous membranes selectively filter compounds, enhancing specificity even for non‐selective sensor elements. Images adapted with permission from ref. [24, 59] Copyright 2023, National Academy of Sciences, and 2018, Elsevier, respectively.

Several artificial sensors already leverage differences in the physicochemical properties of volatiles to tailor the sensor response and help distinguish different molecules. Increasing the spatial separation between a volatile source and sensing elements can accentuate differences between volatiles based on their physicochemical properties; for example, molecules with higher volatility and diffusivity than others in a mixture will reach the sensor at a faster rate, resulting in effective separation. Such a strategy has been implemented by Aizenberg et al., wherein a volatile liquid is dispensed at the bottom of a cuvette; following evaporation and diffusion through the cuvette, the vapors reach the surface of a mesoporous photonic crystal sensor at the top of the cuvette (**Figure** **3**A).^[^ ^24^ ^]^ Adsorption and condensation of the vapor within the sensor pores increases the overall effective refractive index, causing the reflection spectrum to redshift. The time‐dependent reflection spectrum captures the intricacies of intermolecular forces between gas‐phase molecules and the sensor surface, offering a rich source of information.

The incorporation of filters along the gas route allows for shorter pathlengths required to differentiate volatiles, providing chromatographic separation of analytes based on their polarity and that of the filter. This is akin to the action of the porous nasal turbinates and hydrophilic mucus layers, which hinder the transport of less polar volatiles.^[^ ^52^ ^]^ The *TruffleBot* e‐nose employs filters made from materials like activated carbon, felt, or porous metal within the airflow pathways to the sensor.^[^ ^56^ ^]^ These filters partition analytes based on their physicochemical properties and create distinct signal patterns through selective transport. In Graunke et al., fluoropolymer membranes with varying degrees of fluorination, and, therefore polarity, achieve the selective retention of a variety of application‐relevant gases that are typically difficult to separate due to their similar sizes; these include ethanol, acetone, acetaldehyde, carbon monoxide, hydrogen, and others.^[^ ^57^ ^]^ Taylor et al. employ two separate retention columns, one covered with a polar stationary phase and the other with a non‐polar one, effectively going beyond biology's implementation of a single “retentive” medium (the mucus) with a uniform polarity.^[^ ^58^ ^]^ The gaseous sample passes through either one or the other column to one of the two 300‐element sensor arrays, which feature identical sensing elements to produce diverse spatiotemporal responses that are then fed into probabilistic neural networks for classification of the analytes. To differentiate volatiles with even greater specificity, Güntner et al. develop microporous membranes, for example, those based on zeolites, to identify formaldehyde (Figure 3B)^[^ ^59^ ^]^, which is a potential breath marker of cancer and an indoor air pollutant. Formaldehyde is notoriously challenging to detect due to its high reactivity and similarity to other small organic molecules, as well as the need to detect it at particularly low concentrations (<100 ppb) for most applications.^[^ ^60^ ^]^ By tuning the chemistry and porosity of membranes placed in front of the sensing elements, it is possible to achieve remarkable selectivity with otherwise sensitive but highly non‐specific sensors.^[^ ^59^ ^]^ The potential for further improvement lies in dynamic strategies. By adjusting transport pathways in response to real‐time sensor feedback—similar to the way turbinates adaptively filter and condition air—artificial sensors could achieve more selective and efficient analyte detection.

The physicochemical properties of an odorant ultimately affect its binding strengths (i.e., affinities) to different ORs, critical in the production of unique odorant‐OR patterns. This is at the heart of the olfactory system's ability to distinguish even very similar molecules, such as the stereoisomers R‐ and S‐ carvone, which smell like spearmint or caraway, respectively.^[^ ^61^ ^]^ The patterns of binding also enable refined sensing in complex environments; for example, mixtures that only slightly differ in the composition produce overlapping but distinguishable binding “fingerprints”. Further, when components in an odorant mixture saturate some ORs due to a relatively higher binding strength, minor odorant components can still be detected through receptors with lower affinities, as explored, for instance, in fruit flies (*Drosophila melangostar*).^[^ ^62^ ^]^

These interactions are often further fine‐tuned by proteins present in biological olfactory systems that can directly interact with odorants to increase or reduce their concentrations at the ORs. Water‐soluble odorant binding proteins (OBPs) found in the mucus of many organisms from insects to mammals aid in the transport of hydrophobic molecules across the hydrophilic mucus layer and release them near the ORs.^[^ ^63^, ^64^, ^65^, ^66^ ^]^ Additionally, in dynamic environments, where real‐time odor sensing is critical, as in predator–prey scenarios, a rapid turnover rate of odorant binding is essential. In most mammals, this can be achieved through exhalation that forces the desorption of the odorant from the OR, or through the degradation of the odorant molecule, potentially via xenobiotic metabolizing enzymes (XMEs).^[^ ^51^, ^67^, ^68^, ^69^, ^70^ ^]^ The combined action of OBPs and XMEs in the mucus layer of the olfactory epithelium introduces an additional level of control over odorant transport, allowing for both reliable sampling of the environment and the fine‐tuning of detection toward specific chemicals of interest.

Mimicking the function of these enzymes has yet to be fully realized in e‐noses. Current methods for desorbing molecules from e‐noses, such as passive diffusion, forced airflow, or thermal desorption (e.g., TCO), are largely non‐selective and typically remove only physically adsorbed molecules, leaving behind residue buildup that can degrade the sensor elements over time. This accumulation, along with poisoning and other sources of damage from the regular operation of a sensor, often leads to sensor drift. Incorporating catalytic molecules that mimic the role of XMEs to break down and eliminate even trace amounts of both physically and chemically adsorbed molecules could effectively prevent this buildup, thereby preserving the sensor's accuracy and extending its operational life. Such strategies underscore the potential of optimizing the sensor output, based on the physicochemical properties of target volatiles, whether by accentuating or mitigating specific molecular classes, and provide a promising framework for advancing artificial gas sensing technologies.

## 4 The Olfactory Bulb and Signal Integration

**Figure 4:**
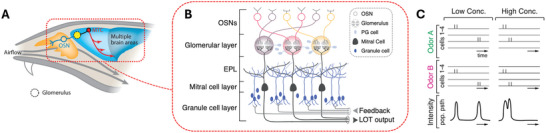
The neuronal pathways of the olfactory system. A) Odorants interact with ORs, creating distinct binding patterns across several ORs rather than a single strong binding. These patterns are transmitted by OSNs to the brain via mitral and tufted cells (MTC). (B) OSN axons converge in specific glomeruli within the olfactory bulb, where the spatial organization of these inputs aids in odor discrimination. Mitral and tufted cells then relay this information to higher brain regions, such as the piriform cortex, for further processing. PG cell: periglomerular cell; LOT: lateral olfactory tract; EPL: external plexiform layer. C) Temporal dynamics in the piriform cortex show that stronger OR‐odorant interactions result in earlier neuronal firing, while weaker interactions contribute to delayed signal transmission, with timing influenced by odorant concentration. Images adapted with permission from ref. [72, 78, 79] Copyright (2022) Annual Reviews, (2020) Elsevier, and (2020) Elsevier, respectively.

The Nobel Prize‐winning discoveries by Linda Buck and Richard Axel of a gene family encoding ORs, and the subsequent description of odor‐sensing mechanism based on patterns of binding of odorants to large numbers of ORs were revolutionary to the fields of biology and sensing, shedding light on the mystery of how a relatively small number of receptors can identify and distinguish orders of magnitude more odorants.^[^ ^71^ ^]^ The ORs, part of the G protein‐coupled receptor family, are situated on protrusions on the dendritic (signal‐transduction) side of olfactory sensory neurons (OSNs), which span the olfactory epithelium. When an odorant binds to an OR, it triggers a biochemical cascade that ultimately generates action potentials, which is then relayed via the OSN axons (signal‐transduction side of neurons) to deeper regions of the brain, such as the piriform cortex, to be processed and analyzed (**Figure** **4**A).^[^ ^71^, ^72^, ^73^ ^]^ As each odorant binds to multiple ORs with varying strength, it produces a binding pattern or “fingerprint” of action potentials. It is this recognition and processing that enables mammals, having on the order of hundreds to a thousand putative OR genes, to distinguish orders of magnitude more odorants and their mixtures.^[^ ^51^, ^74^ ^]^

To facilitate signal interpretation, the collection of action potentials emanating from different OSNs is encoded spatiotemporally.^[^ ^75^ ^]^ The spatial encoding is derived from the organization of the OSNs: though they are distributed quite uniformly on their dendrite side, with no apparent clustering by type, their axons converge at specific regions in the olfactory bulb known as glomeruli, i.e., OSNs displaying the same OR have their axons cluster in the same glomerulus (Figure 4B).^[^ ^76^, ^77^, ^78^ ^]^ The signal is further transmitted by mitral and tufted cells, often called “projection cells”, to various parts of the brain via their axons, which together make up the lateral olfactory tract. Here, the signal encoding becomes quite complex and continues to be actively investigated, with the information likely being non‐linearly encoded. Temporal encoding is evident in the piriform cortex, the brain region critical to identifying odorants and their concentrations.^[^ ^75^, ^79^ ^]^ Indeed, relatively high‐affinity odorant–OR binding events trigger neuronal activity in the piriform cortex typically between 50–100 ms after the binding event and is largely independent of the odorant concentrations. In contrast, action potentials from lower‐affinity binding events happen after this initial peak, with their latency decreasing as the concentration of the odorant molecules increases (Figure 4C).^[^ ^79^, ^80^, ^81^, ^82^, ^83^, ^84^ ^]^

The strategy of spatial distribution and unit replication of the electronic sensor to better classify target analytes, akin to the OSN in mammals, has been adapted in e‐noses. Kang et al. utilized glancing angle deposition to fabricate a gas sensor array composed of nanocolumnar films of four different metal oxides. In testing, the sensor array, containing four replicates of each sensor material, demonstrated high uniformity in resistive response (27.07 kΩ ± 4.03 kΩ).^[^ ^85^ ^]^ Combined with a time‐varying sensor response, this device demonstrated high‐accuracy and real‐time detection of multiple gases. The spatial and temporal neuronal firing patterns observed in the olfactory bulb and piriform cortex can be effectively mimicked in artificial sensors, where neural networks or other ML algorithms serve as the “brain” for data relay and analysis. For instance, an odor *j* interacts with the active material of a sensor *i*, the chemical input is converted into a time‐dependent voltage signal *v_ij_* (*t*), where *t* needs to exceed the minimum time for acquiring reliable information. As the number of sensor elements or the duration of data collection scales up, the dimensionality of the interpretable dataset increases accordingly. This high‐dimensional dataset is subsequently mapped to a known set of odor descriptors (*k* classes), that can be used to improve the classification and the identification of unknown odorants in more complex scenarios.

Many e‐nose papers employ support vector machines (SVMs), random forests, k‐nearest neighbors, etc., to identify signature patterns in sensor data.^[^ ^86^ ^]^ While these algorithms are effective at handling basic odor classification tasks, they struggle with high‐dimensional, time‐resolved data.^[^ ^7^, ^87^ ^]^ As the complexity of the data increases, the “distance” metrics used to differentiate between odors (treated as classes in ML) begin to collapse, leading to diminished accuracy, particularly when dealing with a larger number of target gases or mixtures.^[^ ^88^ ^]^ Starting in the 1980s, more sophisticated models using algorithms such as Recurrent Neural Networks and their specialized variant, long short‐term memory networks, have been developed to handle sequences of data over time. These models expand on hidden Markov models (HMMs), which utilize a series of nodes all connected with each other in a chain.^[^ ^89^ ^]^ In this framework, each subsequent observation (node) is based not only on its independent probability of occurrence (the prior distribution) but also on the evolving information from previous time points (the posterior probability). This allows for a more nuanced understanding of temporal relationships within the data. Convolutional neural networks, though traditionally used for spatial relationships in image data, have been cleverly adapted to handle time‐sequenced data by transforming temporal relationships into spatial ones, such as through the use of river plots to train residual network models.^[^ ^90^ ^]^ The continuous and accelerating evolution of these models has led to the development of transformer networks, which are now state‐of‐the‐art for encoding complex relationships over longer time horizons enabling contextual data from a distant past to be recalled and used alongside new sensor information.^[^ ^91^ ^]^ These advanced neural networks will be pivotal for both the detection and quantification of gases present in the environment.

Building on this momentum, ML has also shown great promise in the realm of chemical structure identification, particularly through quantitative structure–activity relationship (QSAR) models,^[^ ^92^, ^93^, ^94^ ^]^ and more recently quantitative structure–odor relationship models.^[^ ^95^ ^]^ These models utilize cheminformatics–inferring molecular properties, such as scent, from a molecule's structure, thus enabling the prediction of olfactory profiles based on chemical signatures.^[^ ^95^, ^96^, ^97^ ^]^ Recent work has expanded on this by using QSAR to predict the interactions between airborne chemicals and sensor arrays^[^ ^98^, ^99^ ^]^, linking chemical structure to sensor responses that may enhance the accuracy of scent detection and classification. More commonly, QSAR models (and invertible deep neural networks) have been employed to map sensor data back to specific chemical structures (e.g., among *k* finite classes).^[^ ^100^, ^101^, ^102^ ^]^ This opens the door for advanced methods of chemical identification and profiling from complex sensor outputs. Such techniques could further optimize artificial sniffing systems by predicting the chemical composition of environmental volatiles from the signal they induce, streamlining both detection and classification.

Robust data processing techniques are crucial to the implementation of the ML models. Time integration, using methods such as signal packaging and signal integration, plays a key role in enhancing the reliability and quality of datasets generated by artificial sensors. Signal packaging places the signal in well‐defined units, an encoding of the olfactory “perceptual moment,” meaning that signals are integrated over a specific time duration to form a cohesive perception of the odor. Such integration happens for all senses; for vision, for example, this moment is ≈50 ms, meaning that all contextual information within each of these moments is averaged and likely processed as a singular package of information by the brain.^[^ ^103^ ^]^ Note that this is different from the concept of latency and reaction time, in which changes in the visual perception can trigger at different rates compared to the perceptual moment.^[^ ^104^ ^]^ In artificial sensors, signal packaging is achieved by implementing various time‐based filters (i.e., moving average, Kalman, particle), ensuring that noise is removed during the integration period while the signal remains intact. Such data processing is widely implemented in artificial sensors but can be further improved by identifying and utilizing optimal time‐windows—the packaging window—more closely considering the underlying physical mechanisms, including the binding and sorption kinetics, as well as transport dynamics. This could achieve higher SNR, enhancing the accuracy and reliability of sensor data before it is processed by ML models.

Another facet of time integration involves short‐term memory, where the currently active sensors are influenced not only by the present chemical environment but also by the lingering effects of recent past exposures. This allows for more accurate odor identification by capturing and processing the data with temporal context rather than simply the last package of information. For instance, Schleif et al. demonstrated improvement in the identification of analytes when temporal sensor data was used to train a Supervised Generative Topographic Mapping Through Time (SGTM‐TT) model–another example with HMM at its core.^[^ ^105^ ^]^ Compared to SVM and other models that cannot handle time‐sequences gathered under operation in the field, SGTM‐TT showed a remarkable improvement when it considered increasing time horizons from 1 to 10 s; however, in their case, this improvement does not extend to longer time‐windows (e.g., 20 s) since most of the chemical information is captured in the transient parts of the signal (rise and decay) rather than the noisy steady‐state equilibria.

While researchers have begun implementing software solutions that mimic how the brain interprets olfactory data, there is much more to be learned from the olfactory system's neuronal circuitry. For instance, the role of granule cells, which send inhibitory signals to mitral and tufted cells, serves as an early form of signal processing and attenuation, as highlighted in recent research.^[^ ^106^ ^]^ This biological process is crucial in preventing the olfactory system from becoming overstimulated by non‐lethal and static environmental scents, essentially re‐calibrating itself dynamically to focus on changes rather than static stimuli (i.e., through the first derivative of the signal). By applying this lesson to sensor design, we can move beyond current practices that rely heavily on signal recalibration, deep neural network models, or sensor replacement.^[^ ^25^, ^107^, ^108^ ^]^ For example, incorporating proportional‐integral‐derivative (PID) controllers, decoupling capacitors, high‐pass filters, and differential amplifiers could allow sensors to dynamically adjust and attenuate non‐changing signals, much like how the olfactory system re‐baselines itself over time. Moreover, the concept of signal deconvolution remains underutilized in current sensor array systems. In nature, each OR not only binds to a range of molecules but also outputs a convoluted signal that contributes to a collective understanding of the environment. The mystery of proper signal deconvolution without the use of “black box” ML approaches has limited the design of gas sensor circuits that leverage this behavior. Yet, emerging work by Mathis et al. on a linear decoder that can directly predict target odors from glomerular inputs provides a framework for designing electronic circuits to mimic the complexity of olfactory bulb neural circuits.^[^ ^109^ ^]^ We envision that researchers could design systems where multiple sensors work together within interconnected domains, enhancing the ability to distinguish between complex gas mixtures, similar to how biological systems differentiate odors by processing overlapping signals. This approach can be hierarchical in that these domains themselves are arrayed and integrated into dual‐nostril‐inspired systems, which encode fluid dynamics unique to each sensor array in a manner similar to how nostrils in mammals provide enhanced spatial and chemical information.

In summary, biology has illuminated four key methods for signal processing that can greatly enhance sensor technology: signal packaging, which captures the perceptual moment; signal integration, akin to short‐term memory; signal deconvolution, enabling spatial or sensorial de‐mixing; and signal recalibration through inhibitory feedback. By implementing these biologically inspired innovations together with emerging discoveries related to biological olfactory signal processing, we can significantly improve the speed and accuracy of sensor arrays, ultimately providing more detailed and real‐time insights into complex environments.

## 5 Active Sniffing to Mimic Natural Analyte Transport

**Figure 5:**
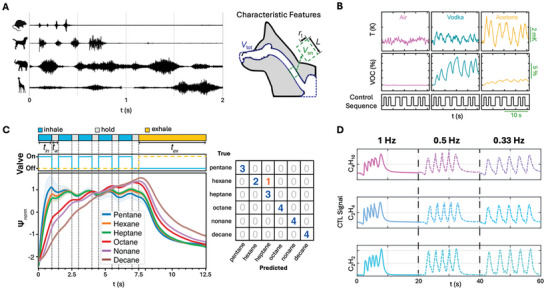
Sniffing as a key element in natural and artificial olfaction. A) In biological systems, deliberate sniffing enhances odor detection by actively modulating airflow to optimize odorant delivery to the olfactory epithelium, as seen in animals like dogs. B) Artificial systems can mimic this by controlling airflow and environmental factors, such as temperature and humidity, to improve sensor performance. C) Active controlled sniffing sequences in artificial systems have been shown to enhance detection accuracy and reduce response times. D) Reinforcement learning models hold promise for adaptive sniffing strategies, optimizing sniffing patterns in real‐time based on analyte concentration and environmental changes, much like how animals adjust their sniffing frequency. Images adapted with permission from ref. [22, 23, 24, 115] Copyright 2021, Nature Portfolio, (2020) IEEE, (2023) National Academy of Sciences, (2024) Wiley‐VCH, respectively.

Sniffing is a critical process in biological olfaction, involving deliberate variations in inhales, holds, and exhales to improve odorant detection and discrimination, particularly aiding in the detection of volatiles at low concentrations to enhance the delivery of odorants to the olfactory epithelium. The active control of airflow also allows fine‐tuning of the interactions between analytes and the ORs, providing additional information for odor discrimination and enhancing olfactory effectiveness across diverse environments. Beyond more frequent environmental sampling, varying sniffing patterns potentially change the relative concentrations of odors at ORs, allowing the organism to refine its understanding of the olfactory landscape through “multiple overlapping sniffs”, including filtering out a “static” background.^[^ ^110^ ^]^ Sniffing also appears to be correlated with firing patterns of mitral and tufted cells as well as the olfactory bulb more broadly and sniffing undoubtedly plays a vital role in navigating complex odor landscapes.^[^ ^111^, ^112^ ^]^ Animals like canines and rodents adjust their sniffing frequency and intensity in response to various odor sources, enabling them to sample a wide range of odorants more effectively, which is crucial for tasks such as tracking scent trails or locating food (**Figure** **5**A).^[^ ^113^ ^]^ Non‐mammalian species have also developed adaptations to actively modulate the flow of chemicals in their environments, thereby providing additional information to their neural circuitry, including enhanced detection efficiency (by effectively increasing the concentration of chemicals) and spatial resolution. For example, crustaceans like the blue crab (*Callinectis sapidus*) flick their antennules while many insects flutter their antennae during flight to enhance airflow and improve the detection of pheromones.^[^ ^114^ ^]^

To apply sniffing to e‐noses, we consider the efficiency of olfactory detection, defined as the resolvable information per unit time (i.e., the number of molecules available to the sensor per second). This has been shown to be influenced by the characteristic time and length scales of sniffing, as well as dimensionless numbers like the Womersley number (Wo), which relates sniffing frequency (f) and the kinematic viscosity of air (ν) through the relationship:

(1) $$Wo=D_{H}\sqrt{\frac{\pi f}{2\nu }},\;\;\;\;\;D_{H}=\frac{4A_{C}}{P}$$

where *D_H_*, *A_C_*, and *P* represent the hydraulic diameter, sensing surface area, and the perimeter of a cross‐sectional area, respectively. As sniffing frequency rises, Wo increases, leading to greater information gain per unit time which has been shown, for example, by Spencer et al., demonstrating that when mice and rats increase their sniffing frequency by ≈75% in the presence of an odor source, there is a corresponding 10% increment in information encoded per unit time.^[^ ^115^, ^116^ ^]^ However, an increase in sniffing frequency also led to a continuous reduction in signal amplitude decreasing the SNR, potentially to limits where detection becomes impossible. To mitigate this, Kennedy et al. explored varying sniffing frequencies to maintain signal integrity in turbulent gas flows.^[^ ^117^ ^]^ In their system, consisting of a long cylindrical chamber equipped with PID sensors and computer‐controlled solenoid valves, they found that adjusting sniffing frequency in proportion to the feeding flow velocity of a gas sample can enhance information preservation, with a specific optimal frequency that maximizes information transfer unique to each sensor–analyte system.

Similar to natural systems, e‐noses can harness the principles of sniffing to enhance the range and quality of physicochemical information gathered.^[^ ^118^ ^]^ Unlike their biological counterparts that can only modulate fluid flow, artificial sniffing can also, to a larger degree, control environmental parameters like temperature and humidity—key factors that influence the pseudo‐chemical equilibrium of each chemical interacting with the sensor. By imposing specific patterns of air intake (“inhaling”) and expulsion (“exhaling”), along with controlled dwell times (“hold”), artificial sniffing can modulate the concentration of volatiles adsorbed to the sensor surface and adjust their kinetics and equilibria, directly impacting sensor output (Figure 5B).^[^ ^23^ ^]^ For example, Aizenberg et al. demonstrated that variations in artificial sniffing sequences affected multiple volatiles differently due to variations in their interaction strengths with sensor surfaces.^[^ ^24^ ^]^ By implementing active sniffing through solenoid valves in the gas delivery path, they improved the classification accuracy of volatile compounds from 88% to 95% and reduced the classification time from 600 s to just 12.5 s, underscoring the potential of controlled sniffing patterns to refine sensor performance (Figure 5C).^[^ ^24^ ^]^ This quicker detection and improvement in performance is sensor‐agnostic as it has also been observed for chemiresistive arrays and emerging cataluminescence‐based sensors.^[^ ^22^, ^23^ ^]^

Another critical design consideration for artificial sniffing systems is ensuring that the desorption time of odorants is shorter or comparable to the sniffing frequency. For instance, in photonic crystal sensors, where saturation typically occurs within 1–5 s, tuning the sniffing frequency to match these kinetics ensures effective odorant binding and desorption cycles, thereby enhancing sensor performance.^[^ ^24^ ^]^ Sensor technologies that incorporate active sniffing show clear benefits, including improved accuracy, faster response times, and the capacity to handle complex odorant profiles. As the understanding of odorant–sensor interactions deepens, the strategic implementation of sniffing in artificial systems will likely continue to advance, offering new opportunities for sensor technology development.

Despite the advancements provided by prescribed sniffing patterns, a significant challenge remains in achieving free‐form sniffing that self‐optimizes and adapts dynamically to varying environmental conditions. To address this, we propose the use of reinforcement learning (RL) as a promising avenue for the further enhancement of sensor performance. Unlike traditional ML models that predict outcomes based on historical data, RL models operate on a reward‐based system, where the sensor system iteratively refines its sniffing strategies by receiving rewards or penalties based on the effectiveness of its actions. Through continuous interaction with the environment and adjusting its behavior to maximize cumulative reward, the RL model learns to optimize sniffing sequences in real‐time, thereby making more informed decisions and adapting to changing conditions more effectively. As these models do not require the notion of ground truth, the formulation of the reward functions is a topic of investigation both in the research and engineering communities. For example, the effectiveness of an action can be judged by the lowering of uncertainty in the classification task, but at the same time, a penalty can be charged for both taking longer to do so but also, akin to biological systems, measuring the expenditure of energy (i.e., over‐switching of actions or fast sniffing). This approach opens up exciting new possibilities for artificial gas sensing by using RL models to mimic living organisms by adjusting airflow and frequency based on real‐time sensor data. This adaptability helps the system optimize each sniffing cycle to gather the most information. For example, an RL model could learn to change sniffing frequencies depending on analyte concentration (Figure 5D).^[^ ^22^ ^]^ At lower concentrations, slower sniffing—similar to a deep breath in animals—can increase the chances of binding events and improve detection limits. In contrast, at higher concentrations, faster sniffing speeds up data collection, leading to quicker identification of compounds. Modulation of the sniffing pattern could also help distinguish novel events from a constant background. Incorporating characteristic material information or analytical modeling into sniffing pattern determination may further improve classification and concentration estimation accuracy. For example, sorption kinetics parameters derived from analytical model fitting have proven effective for discriminating 20 distinct odors using an array of nanomechanical sensors.^[^ ^119^ ^]^ Similarly, when paired with a classifier, an RL agent could optimize sniffing frequencies to highlight the unique sorption kinetics of compounds that remain uncertain to the classifier.^[^ ^120^ ^]^ By dynamically adjusting sniffing patterns, the RL model can improve classification accuracy and enhance the detection of specific compounds under varying environmental conditions. Additionally, incorporating multiple “nostrils” with different flow rates, each connected to similar sensor elements, could modulate flow patterns and provide a more detailed understanding of the environment. This setup would not only enhance detection accuracy but also improve spatial awareness, allowing the system to distinguish between different odor sources and their locations. This improved spatial awareness would be especially useful in complex environments with moving sources or changing wind patterns, helping the sensor system navigate and identify specific sources more effectively.

## 6 Motor Responsiveness for Chemotaxis and Spatial Awareness

**Figure 6:**
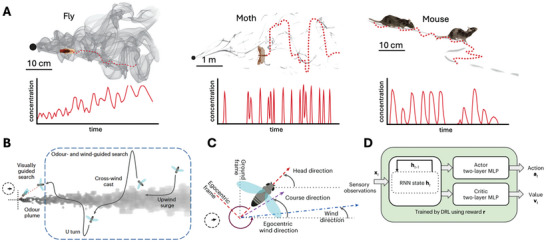
Chemotaxis and casting behavior in natural and artificial systems. A) Animals like flies, moths, and mice use casting—zig‐zagging motions with varying amplitude—to track odor sources, adjusting search strategy dynamically based on scent concentration and uncertainty. B) Flying animals exhibit oscillations, switching between crosswind and upwind paths to locate prey or other odors via olfactory cues. C,D) Robotic systems, equipped with chemical sensors and reinforcement learning, can mimic these behaviors for gas plume detection and tracking. Images adapted with permission from ref.^[^ ^72^, ^128^ ^]^ Copyright (2022) Annual Reviews, 2023, Nature Portfolio, respectively.

Many organisms display chemotaxis—movement toward a chemical stimulus, such as an odor—or its opposite, negative chemotaxis, both directed by spatial gradients of stimulus concentration.^[^ ^121^, ^122^, ^123^, ^124^, ^125^ ^]^ Although the precise neurological mechanisms governing odor tracking remain only partially understood, especially in insects and larger organisms, certain common behaviors have been observed. One of them is the “casting,” where the animal oscillates around a perceived odor trail.^[^ ^122^, ^126^, ^127^ ^]^ The amplitude of these oscillations is thought to be linked to the animal's confidence in the scent direction, with wider sweeps occurring when the scent path is broken or uncertain (**Figure** **6**A).^[^ ^72^ ^]^ Interestingly, studies in casting rats have shown that their movement frequency closely matches the optimal sampling frequency predicted by the Shannon‐Nyquist theorem (≈2.85 to 2 Hz), suggesting a highly efficient strategy for gathering sensory information.^[^ ^122^ ^]^ Similar oscillatory movement patterns are seen in flying animals like fruit flies (*Drosophila melanogaster*), gypsy moths (*Lymantria dispar*), and wandering albatrosses (*Diomedea exulans*) as they track odor sources (Figure 6B).^[^ ^125^, ^128^, ^129^, ^130^ ^]^ For example, albatrosses often follow a crosswind flight path before switching to an upwind, oscillatory pattern once the prey is detected. This behavior contrasts with the wind‐independent trajectory that would be expected if the prey were located purely by sight, highlighting the importance of olfactory cues in spatial awareness and motor response.^[^ ^130^ ^]^

Advances in robotics and aviation over recent decades have led to the development of numerous automated quadruped units and drones that are now commercially available for various real‐world gas sensing applications. These include search and rescue operations, environmental monitoring, infrastructure inspection, and precision agriculture, all of which rely on sensor‐driven motion feedback. Notable examples are the robotic dogs created by Boston Dynamics and Ghost Robotics, as well as drones from Sniffer Robotics and Mine Kafon.^[^ ^131^, ^132^, ^133^, ^134^ ^]^ The integration of chemical sensors into these systems opens up new possibilities, such as detecting explosive or chemical threats, automating inspections, and enhancing search and rescue efforts.^[^ ^135^, ^136^, ^137^ ^]^ For tasks that require precise localization of gas sources, incorporating chemotaxis‐based navigation can significantly enhance accuracy (Figure 6C) *(128)*. Devices equipped with anemometers or barometers can measure local wind direction, enabling them to mimic the crosswind flight pattern used by albatrosses to locate gas plumes. Once a plume is detected, a robotic system can switch to the efficient “casting” behavior to quickly zero in on the gas source.^[^ ^138^ ^]^ This approach was successfully demonstrated by Reddy et al., who trained an RL model to replicate the zig‐zag and casting movements of odor‐tracking rats in a simulated environment.^[^ ^126^ ^]^ Their model minimized the risk of losing the scent trail while optimizing speed along the path, naturally reproducing the behavior of widening casting sweeps when the trail was lost. In robotic systems, this approach can be further enhanced by integrating data from computer vision, terrain scanning, and chemosensors to optimize artificial scent tracking using RL models (Figure 6D).^[^ ^128^ ^]^

Living organisms offer a powerful blueprint for integrating sniffing behavior with chemotactic responses in artificial systems, mirroring how animals process odors in stereo. For instance, rats and humans use bilateral nostrils to enhance scent tracking, a process that has been studied in depth, particularly in the latter.^[^ ^122^, ^139^ ^]^ Similarly, bilateral odorant detection has been observed in species like fruit flies (*Drosophila melanogaster*), honey bees (*Apis mellifera*), and American lobsters (*Homarus americanus*), which use antennae as their olfactory input points.^[^ ^140^, ^141^, ^142^, ^143^ ^]^ Replicating this in artificial systems involves incorporating two or more gas inlets, allowing each to feed into a sensor or sensor array independently. This approach has been used in some sensor systems for better source localization,^[^ ^138^, ^144^, ^145^ ^]^, but combining it with sniffing patterns for spatiotemporal signal analysis adds a new layer of functionality. This is especially useful in cases where movement is limited, thus the probing of various plume patterns and concentration gradients (streamlines) that cannot be achieved strictly though sensor movement (e.g., casting behavior) or dual nostrils (i.e., differential input to nostrils) can be done so with the fluid flow created by sniffing patterns. Crucially, the combination of these methods doesn't require sensor duplication; rather, data from each inlet can be labeled and processed separately, allowing for more accurate and efficient downstream analysis. Beyond just sniffing, incorporating movement of the sensor apparatus itself—whether in a natural or robotic system—can further enhance odorant tracking by reducing the directional noise introduced by turbulent transport. However, for this to be effective, the movement must span distances greater than the system's turbulence length scale. When these criteria are met, this technique will significantly improve the sensor's SNR, making it a valuable tool for advancing real‐time chemical detection.

## 7 Analyte Chaperones, Sensor Priming, and Odor Complementarity

Detecting the presence of specific target molecules within a mixture is challenging, largely stemming from the range and complexity of interactions between the volatiles themselves and between the volatiles and sensor surfaces. Indeed, one molecular species can promote or inhibit the binding of another in ways that may vary over time, depend on the presence of additional molecules and other environmental factors, and even involve chemical reactions (e.g., ozone can oxidize hydrocarbons like limonene, producing an entirely different sensor response); additionally, water from the air can adsorb to the surface, creating a surface layer over time, affecting volatile binding in a time‐dependent manner.^[^ ^146^, ^147^ ^]^ In the previous sections, we discussed biology's approaches to increase its discriminatory power, such as physically increasing the concentration of target molecules at the OR by implementing designs that control fluid transport and make use of the molecule's unique physicochemical properties. We also examined how binding interactions can be tuned and the neuronal processes involved to maximize the contrast between molecules. In this section, we postulate that natural olfactory systems may also use—rather than be hindered by—codependent interactions between volatiles, including odorless ones.^[^ ^148^, ^149^ ^]^

Synergistic interactions, wherein the presence of one odorant increases the perceptibility of the other or the exact opposite—antagonism or modulation—can be achieved in nature through 1) cooperative or competitive binding of odorants to ORs and 2) modulation of the activity of neurons en route to and in the olfactory bulb. As such effects are essentially unavoidable, we propose the use of molecules that can act as analyte chaperones or participate in sensor priming. These molecules may be chemically identical but differ in their timing of introduction relative to the unknown gas: while chaperones are introduced concurrently with the gas to be sampled, primers are added to the sensor beforehand, establishing a baseline response that is deliberately distinct from the equilibrium response. One example is the use of water vapor. While humidity notoriously confounds sensor readings^[^ ^150^ ^]^, it can be strategically tailored to act as a chaperone or primer.^[^ ^14^ ^]^ For example, several groups have shown that water adsorbed to a sensor surface has an enhancing effect in the detection of ammonia, as it promotes ammonia dissolution and, therefore, interaction with the sensor surface.^[^ ^151^, ^152^, ^153^ ^]^ Water can also act as a “chaperone” for hydrophilic molecules, creating a sharp contrast with hydrophobic molecules, that can be sensed more precisely. Such controlled perturbations in chemical affinity and binding kinetics can reveal subtle features in the sensor array's response, improving detection accuracy. In the perfume industry, for example, it has been anecdotally reported that certain compounds like hedione (methyl dihydrojasmonate) can amplify the perception of other fragrant molecules, particularly with its high‐cis form. Similar synergistic effects have been observed in olfactory studies involving honey bees (*Apis mellifera*), yellowjackets (*Vespula maculifrons*), and other organisms.^[^ ^154^, ^155^ ^]^ Such chaperone effects suggest a broader potential to enhance the detection of other volatiles.

Further exploration into similar phenomena is essential, as the presence of organic chaperones may alter the sorption and desorption kinetics in artificial sensors, thereby modulating their overall performance. Advances in understanding primary odor maps could help identify optimal priming and chaperone molecules^[^ ^97^, ^156^ ^]^, paving the way for more precise and effective gas‐sensing technologies. Despite these benefits, challenges like excessive concentrations of chaperone molecules, condensation of less volatile compounds, or reactions with the chaperone gas can complicate detection. The concept of odorant complementarity, where a specific chaperone molecule reduces uncertainty about the presence of a gas, presents a promising but underexplored strategy in artificial sensing systems. Unlike biological systems, which must balance nasal humidification for respiratory health^[^ ^29^ ^]^, artificial systems are free to experiment with a broader range of chaperones, primers, and carrier gases.

## 8 Challenges and Prospects

Current e‐noses have made substantial progress in artificial chemical sensing, already finding applications across diverse fields. With advances in sensor arrays, pattern recognition, and machine learning models, these devices have become more accurate, compact, and affordable, enabling chemical identification in specific environments. The incorporation of environmental metadata, such as temperature and humidity, has further refined their performance, making them versatile in fluctuating conditions. These achievements have positioned e‐noses as valuable and promising tools for industrial safety, environmental monitoring, and beyond.

**Figure 7:**
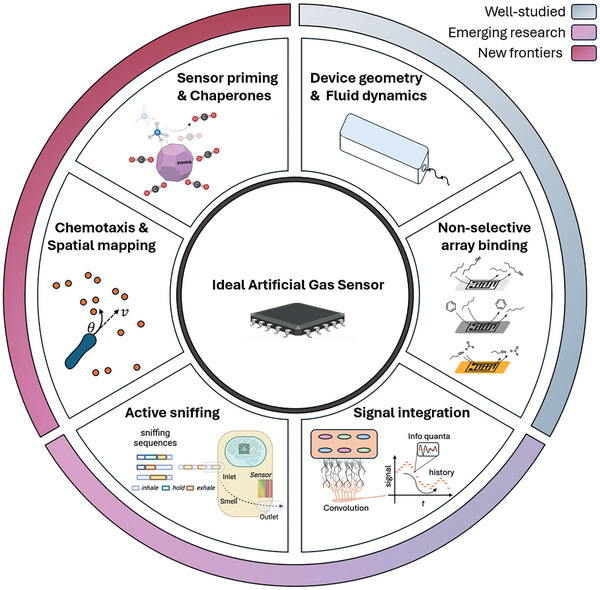
The path to universal, reliable e‐noses mirrors the evolution of biological olfaction, combining well‐established principles—fluid dynamics, device geometry, and non‐selective but speciated sensor materials—with emerging concepts like signal integration and active sniffing, and pushing into new and understudied frontiers, such as chemotaxis, spatial mapping, sensor priming, and analyte chaperones. Integrating the whole palette or a subset of these biological concepts can unlock the full potential of e‐noses, bringing us closer to the real‐world application of e‐noses that rival the remarkable capabilities of natural olfaction.

Incorporating a richer palette of inspiration from the biological olfactory system will move us one step closer to universal e‐nose sensing (**Figure** **7**). For example, optimizing gas transport and composition through geometry adjustments, free‐form and self‐optimizing sniffing sequences, and sensor movement via chemotaxis can enhance sampling accuracy. These physical methods can be complemented by chemical techniques like sensor priming—pre‐conditioning sensors before analytes arrive—and the introduction of analyte chaperones in the carrier gas to modulate interactions, which will help improve the detection of specific compounds that are hard to identify in a mixture. Another avenue for improvement in mixture detection lies in tuning sensor–odorant kinetics and equilibria. This can be achieved through temperature‐controlled operations that regulate desorption kinetics, the use of xenobiotic metabolizing enzyme analogs (e.g., catalytic nanoparticles) for complete residue removal, or hardware such as PID controllers and differential amplifiers that mimic inhibitory behaviors like those of granule cells in biological systems, suppressing redundant signals. Additionally, drawing inspiration from how the brain processes signals offers insights into refining signal integration and processing. Techniques like signal packaging for capturing perceptual moments, signal integration for short‐term memory, deconvolution for tracing spatial or sensory pathways, and recalibration through feedback loops can all lead to more efficient and accurate detection. Combining these strategies with dual‐inlet systems (i.e., mimicking nostrils), advanced supervised machine learning models for classification and quantification, and reinforcement learning models for real‐time dynamic adjustments of the sensor system could significantly reduce task uncertainty and extend sensor longevity, offering more robust and adaptive sensing systems. Inspiration can also be sought in the unique anatomy, physiology, fluid dynamics, and chemistry of olfactory systems across the tree of life. Developments in materials with stimuli‐responsive and self‐healing behaviors, as well as the implementation of machine learning capable of adjusting to changes through dynamic self‐optimization and self‐calibration, can bring us closer to achieving the adaptability and plasticity of natural olfactory systems. By increasing the accuracy, sensitivity, specificity, and efficiency of detection, bioinspired designs will enable more compact form factors for e‐nose devices. Together, known and emerging biological principles thus provide a rich source of inspiration for future developments in e‐noses, empowering them to surpass their current limitations.

The future of e‐noses holds great promise, but several challenges must be addressed to fully realize their potential. Achieving advanced capabilities such as sensor priming, xenobiotic metabolizing enzyme analogs, and dual‐inlet systems will require a deeper understanding of gas–sensor interactions, particularly in tuning sensor kinetics and optimizing chaperone molecules without overwhelming signals. Additionally, integrating sophisticated spatiotemporal signal processing, inspired by biological olfaction, will necessitate the development of robust computational models and reinforcement learning algorithms for real‐time adjustments in dynamic chemical environments. Overcoming these obstacles will require interdisciplinary efforts in biology, chemistry, and computational science, along with continuous innovation in both hardware and software. However, the potential rewards are vast, with applications ranging from breath analysis for disease detection and indoor air quality monitoring to agricultural emissions tracking, industrial leak detection, and military applications like landmine detection. Addressing these challenges will enable the next generation of e‐noses to deliver unprecedented precision, adaptability, and reliability across multiple industries.

## Conflict of Interest

The authors declare no conflict of interest.
